# Indoxyl Sulfate Induces Oxidative Changes in Plasma and Hemolysate

**DOI:** 10.3390/molecules27123848

**Published:** 2022-06-15

**Authors:** Anna Pieniazek, Michal Kopera, Lukasz Gwozdzinski, Krzysztof Gwozdzinski

**Affiliations:** 1 Department of Molecular Biophysics, Faculty of Biology and Environmental Protection, University of Lodz, 90-236 Lodz, Poland; michal.kopera@biol.uni.lodz.pl (M.K.); krzysztof.gwozdzinski@biol.uni.lodz.pl (K.G.); 2 Department of Pharmacology and Toxicology, Medical University of Lodz, 90-753 Lodz, Poland; lukasz.gwozdzinski@umed.lodz.pl

**Keywords:** indoxyl sulfate, hemolysate, plasma, oxidative stress

## Abstract

The deteriorating function of the kidneys in chronic kidney disease (CKD) is associated, among other things, with the retention of many unnecessary metabolic products in the body. Indoxyl sulfate (IS) belongs to the group of uremic toxins with a high protein binding affinity. Moreover, this compound can generate oxidative stress. We hypothesized that a high concentration of IS might induce oxidative changes in erythrocytes and plasma components, and could therefore contribute to CKD progression. In this study, we evaluated the influence of IS on the oxidative stress parameters in plasma and hemolysate. Moreover, as a result of the action of IS, we observed a decrease in the total antioxidant capacity and a change in the activity of catalase and superoxide dismutase in hemolysate and plasma. The obtained results indicate that IS induces oxidative damage to hemolysate and plasma components. Greater changes in the parameters of oxidative stress were observed in hemolysate than in plasma treated with indoxyl sulfate. The obtained results suggest that the increased concentration of IS in patients with chronic kidney disease may lead to a decrease in the lifespan of erythrocytes in their bloodstream.

## 1. Introduction

Chronic kidney disease (CKD) is a progressive loss of kidney function associated with the retention of many metabolites in the body. Many accumulated metabolites have toxic properties toward cellular components. Along with the retention of waste products in the body, patients may experience many different ailments related to the occurrence of other pathologies, which significantly reduce the quality of life and may cause excessive mortality. CKD is not limited to the dysfunction of kidneys only. Scientific research provides evidence that high levels of accumulated metabolites called uremic toxins have a significant impact on the development and progression of many other systemic disorders. Among them: cardiovascular damage, endothelial dysfunction, insulin resistance, a tendency to infection and inflammation, anemia, cognitive impairment, and others are mentioned [1].

The encyclopedic list of uremic toxins is constantly changing. In 2003, the European Uremic Toxin Work Group identified 90 different compounds classified as uremic toxins [2]. However, the analyses carried out in the following years resulted in the expansion of the list of uremic toxins by another 56 compounds [3].

The toxins dissolved in urine were classified based on their size and binding properties. Indoxyl sulfate (IS) belongs to the group of protein-bound uremic toxins. This compound arises in the liver during the oxidation and sulfation of indole produced in the gut by the bacteria as a product of tryptophan degradation [4]. The level of this toxin in CKD patients is 60–80 times higher compared to healthy people [2,5]. The elimination of this compound from the body has proved to be an enormous problem. It has been shown that the reduction level of this toxin during hemodialysis is only 30–32% [5]. This is largely due to the affinity of IS for proteins, as 97% of this compound is bound to proteins in the body [5,6]. It has been suggested that this toxin is both a nephrotoxin and a vascular toxin, acting particularly via pro-oxidant mechanisms [7]. Many studies provide evidence that IS induces the formation of reactive oxygen species (ROS) in human renal tubular epithelial cells (HK-2) [8], erythrocytes [9], lymphocytes [10], human umbilical vein endothelial cells (HUVEC) [5], and intestinal epithelial cells (IEC-6) [11]. Typically, the increase in the level of ROS in cells is associated with the occurrence of numerous oxidative modifications of proteins, lipids, and DNA. In cells with elevated levels of ROS, the activity of antioxidant enzymes and the non-enzymatic balance of antioxidant activity may change. Our previous studies have shown that proteins and lipids are modified in the erythrocytes treated with indoxyl sulfate in many ways [12]. Particular attention has been paid to the significant changes in the conformational state of hemolysate proteins and the decrease in the internal viscosity of the erythrocytes treated with IS. It has been shown that approximately 97% of IS in the body is bound to proteins. Studies carried out on the efflux of IS on ROS production by HUVEC in the presence or absence of albumin show significantly lower concentrations of ROS in the presence of albumin [5]. This may suggest that plasma performs a significant role in the antioxidant protection of blood cells.

Under these circumstances, the aim of this study was to investigate the oxidative damage and antioxidant potential of hemolysate and plasma treated with IS. We incubated erythrocytes (RBC) and plasma from healthy donors with IS for 24 h and investigated the carbonyl, amino, and thiol groups in proteins, and TBARS, total non-enzymatic antioxidant capacity, catalase (CAT), and superoxide dismutase activity (SOD).

## 2. Results

Hemolysate and plasma after incubation with indoxyl sulfate for 24 h were tested for several parameters related to the presence of oxidative stress. Two concentrations of IS were used for the tests: 0.2 and 1.0 mM. The conducted studies included changes in the functional groups of proteins in hemolysate and plasma exposed to IS. The level of the carbonyl, amino, and thiol groups in proteins was analyzed.

**Figure 1:**
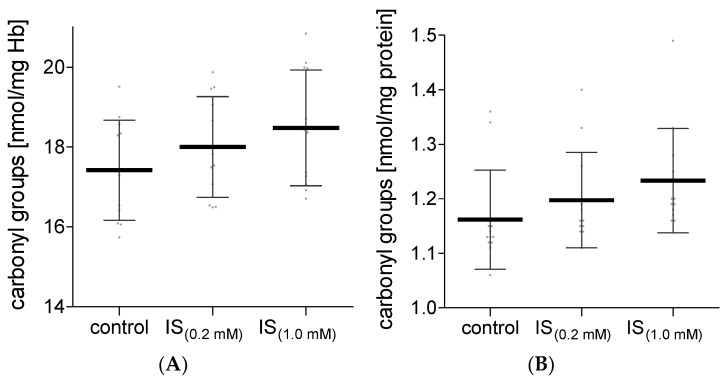
The levels of the carbonyl compound in hemolysate (**A**) and plasma (**B**) after incubation with IS. Data are expressed as mean ± standard deviation, *n* = 12. Gray spots: values of single results.

**Figure 2:**
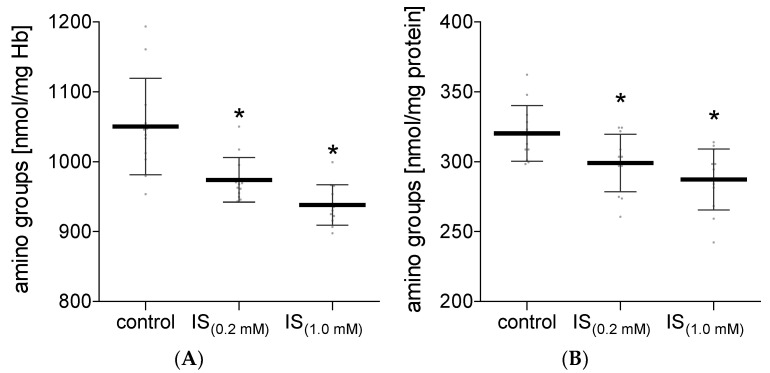
The levels of the free amino groups in hemolysate (**A**) and plasma (**B**) after incubation with IS. Data are expressed as mean ± standard deviation, *n* = 12, * *p* < 0.05—IS_(0.2 mM)_ and IS_(1.0 mM)_ vs. control. Gray spots: values of single results.

The obtained results demonstrated that IS did not affect the concentration of the carbonyl groups in hemolysate proteins, nor in plasma proteins (Figure 1). However, IS caused a significant decrease in the concentration of the amino groups in proteins of hemolysate and plasma (Figure 2). Lower levels of protein amino groups were observed after RBC and plasma incubation with IS at both concentrations used for the experiment in comparison with the control value. However, no significant difference in this parameter was found between the tested IS concentrations.

**Figure 3:**
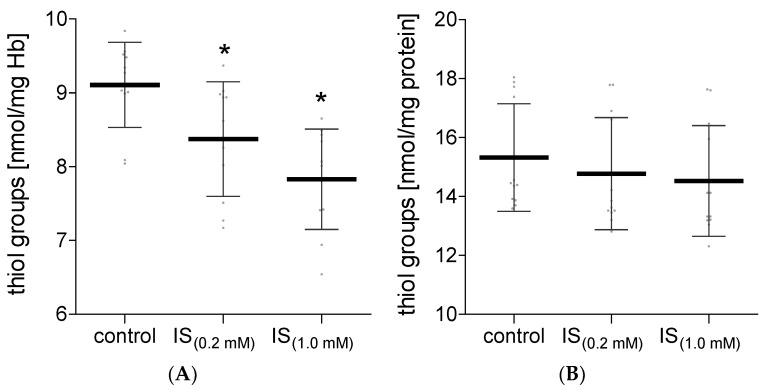
The levels of the free thiol groups in hemolysate (**A**) and plasma (**B**) after incubation with IS. Data are expressed as mean ± standard deviation, *n* = 12, * *p* < 0.05—IS_(0.2 mM)_ and IS_(1.0 mM)_ vs. control. Gray spots: values of single results.

An important element from the perspective of the functioning of proteins and the potential effects of oxidative stress is the level of the free thiol groups in proteins. Our research has shown that IS caused a significant decrease in the level of the free thiol groups in hemolysate proteins (Figure 3A). Lower levels of hemolysate protein thiol groups were observed after incubation of RBC with IS at both 0.2 mM and 1.0 mM concentrations. Surprisingly, IS did not change the level of the free thiol groups in plasma proteins (Figure 3B).

**Figure 4:**
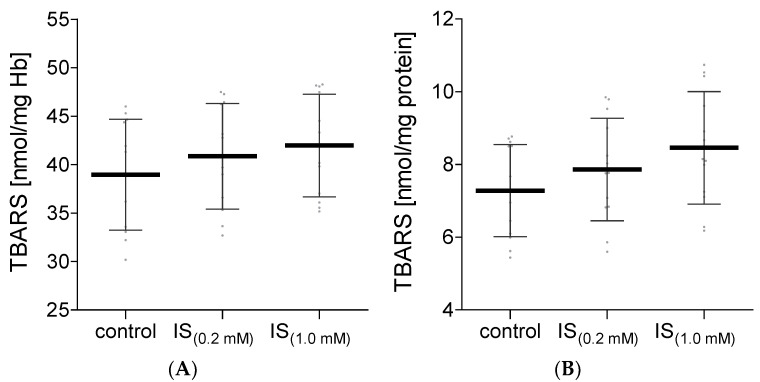
The levels of TBARS in hemolysate (**A**) and plasma (**B**) after incubation with IS. Data are expressed as mean ±standard deviation, *n* = 12. Gray spots: values of single results.

One of the indicators of the induction of oxidative stress in the biological systems is the presence of elevated levels of products that react with thiobarbituric acid. The obtained results did not show any significant changes in this parameter in hemolysate or in plasma treated with IS (Figure 4). It is true that an upward trend in the level of TBARS can be observed along with the increase in the concentration of IS, but these data are not statistically significant.

**Figure 5:**
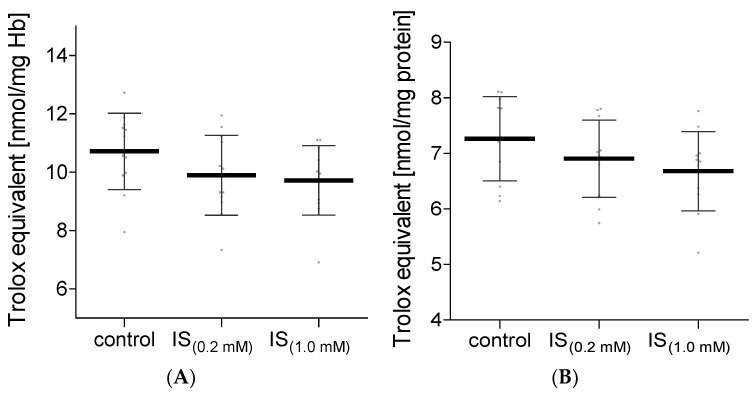
The levels of NEAC in hemolysate (**A**) and plasma (**B**) after incubation with IS, measured using DPPH. Data are expressed as mean ± standard deviation, *n* = 11. Gray spots: values of single results.

**Figure 6:**
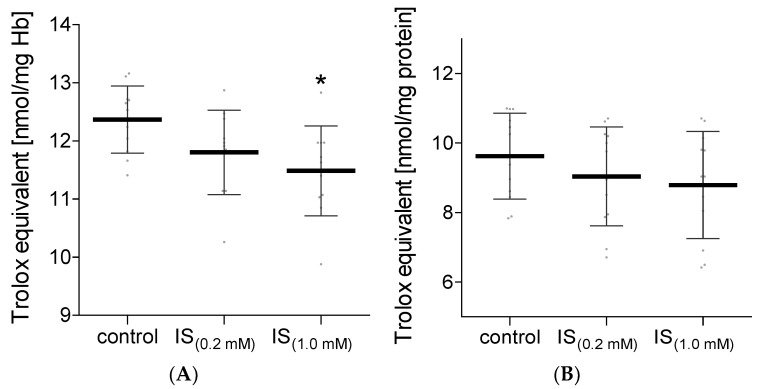
The levels of NEAC in hemolysate (**A**) and plasma (**B**) after incubation with IS, measured using TPTZ. Data are expressed as mean ± standard deviation, *n* = 12, * *p* < 0.05—IS_(1.0 mM)_ vs. control. Gray spots: values of single results.

The increase in oxidizing factors in the biological systems is most often associated with the raised activity of the antioxidant system. The non-enzymatic antioxidant system consists of low molecular weight antioxidants that scavenge ROS. In our study, the total non-enzymatic antioxidant capacity of hemolysate and plasma treated with IS was assessed. Two methods based on different reaction mechanisms were used for this purpose. Measurements of total non-enzymatic antioxidant capacity using the DPPH radical showed no difference in both hemolysate and plasma incubated with IS compared to the control levels (Figure 5). The determination of the level of total non-enzymatic antioxidant capacity using the (Fe [III]-TPTZ) complex in plasma treated with IS also showed no significant differences compared to the control values (Figure 6B). However, this method showed a significant decrease in total non-enzymatic antioxidant capacity in hemolysate exposed to IS at a concentration of 1.0 mM (Figure 6A). In hemolysate treated with IS at a concentration of 0.2 mM, no statistically significant decrease in total non-enzymatic antioxidant capacity was observed compared to the control (Figure 6A).

**Figure 7:**
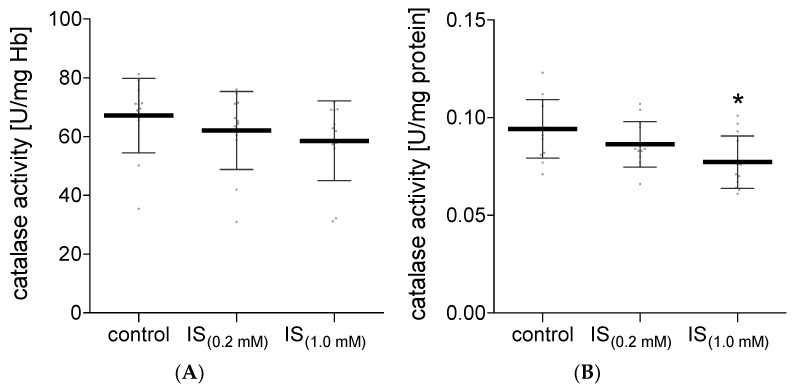
Alterations in the catalase activity in hemolysate (**A**) and plasma (**B**) after incubation with IS. Data are expressed as mean ± standard deviation, *n* = 12, * *p* < 0.05—IS_(1.0 mM)_ vs. control. Gray spots: values of single results.

**Figure 8:**
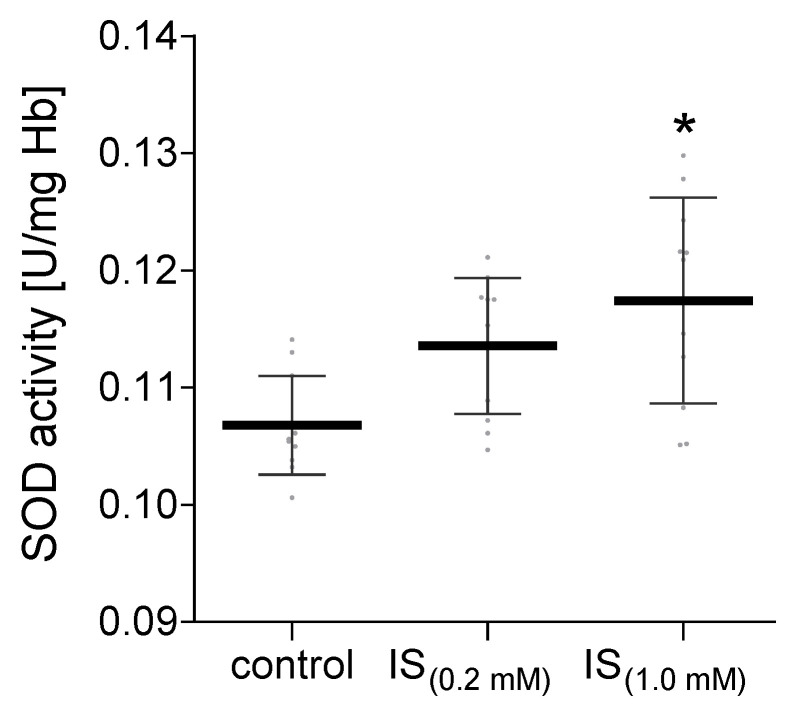
Alterations in the SOD activity in hemolysate after incubation with IS. Data are expressed as mean ± standard deviation, *n* = 11, * *p* < 0.05—IS_(1.0 mM)_ vs. control. Gray spots: values of single results.

The antioxidant enzyme system includes several specific enzymes. In our work, we investigated the activity of CAT in hemolysate and plasma treated with IS. No significant changes in the catalase activity were observed in hemolysate exposed to IS compared to the control (Figure 7A). However, in plasma exposed to IS at a concentration of 1.0 mM, a significant decrease in the CAT activity was observed compared to the control (Figure 7B). In addition to the CAT activity in hemolysate exposed to IS, the activity of SOD was also investigated. It was shown that IS at a concentration of 1.0 mM significantly increased the activity of this enzyme compared to the control (Figure 8). The lower concentration of IS (0.2 mM) had no significant effect on the activity of SOD in hemolysate in comparison to the control (Figure 8).

## 3. Discussion

One of the pathological symptoms of chronic kidney disease is the retention of the waste products physiologically eliminated by the urinary system. Many of these products are toxic and can damage the cell components, and thus contribute to the deterioration of the health of CKD patients. Up to now, more than 145 compounds with this effect have been identified [3]. These compounds are known as uremic toxins. In healthy people, uremic toxins together with IS are usually excreted via the organic anion transport system into the urine [13]. However, in the case of chronic renal dysfunction, toxins accumulate in the blood. This also applies to IS, the concentration of which in the blood of CKD patients may be up to 80 times higher than in healthy subjects [2]. In healthy patients, IS is derived from the breakdown of tryptophan by the microbial community in the large intestine, eliminated by the kidneys, and excreted with the urine. Many of the uremic toxins can be removed from the blood with hemodialysis. However, IS, with its high protein affinity, cannot be disposed of using this method [5]. The toxicity of IS may result not only from its affinity for proteins [14], but also from its ability to generate oxidative stress [9,10,11].

In this work, the effect of IS on oxidative stress parameters in RBC hemolysate and plasma was evaluated. Isolated RBC and plasma from healthy donors were incubated with IS. In patients with CKD, the average concentration of IS in the blood was about 0.2 mM, while the highest observed level was 1 mM. In healthy people, the level of IS is several dozen times lower and averages 0.0024 mM [2]. Hence, these two IS concentration levels were chosen for the research. Oxidative stress accompanies CKD, and the presence of ROS in the plasma of hemodialyzed patients has been demonstrated experimentally [15]. In patients with CKD, a significant decrease in the level of the thiol groups in proteins has also been shown [16,17]. The studies by Pieniazek and Gwozdzinski also showed conformational changes in hemolysate proteins in patients with CKD [16]. Moreover, in our previous work, we showed statistically significant changes in the conformational state of hemolysate proteins, an increase in plasma membrane fluidity, and a decrease in internal viscosity in RBC treated with IS [12]. This result was in accordance with an earlier study that demonstrated that IS could interact with proteins inside erythrocytes. Deltombe et al. indicate that the anion exchanger, Band 3, may be involved in the transport of these toxins through the cell membrane [18]. In our studies, a significant decrease in the levels of the thiol and amino groups in hemolysate proteins upon RBC incubation with IS was determined. Moreover, a slight increase in the levels of the carbonyl groups and TBARS in the hemolysate was also observed. These results suggest that in hemolysate, IS may generate oxidative stress, which can be reflected in the changes of the antioxidant system activity. The redox imbalance in CKD induced by IS has been characterized in detail in previous research [19].

An important element of the antioxidant system, apart from enzymes, are low molecular-weight antioxidants. IS at a concentration of 0.2 mM slightly changed the non-enzymatic antioxidant capacity in plasma and RBC. However, the higher concentration of IS (1.0 mM) in RBC resulted in a significant decrease in this parameter. The main component of the non-enzymatic antioxidant system is glutathione. The studies by Dias et al. showed no changes in the level of glutathione in RBC incubated with IS at a concentration of 0.17 mM [9]. In addition, no significant changes in the level of glutathione in HUVEC cells, after incubation with IS in a similar concentration range, were observed by Duo et al. [20]. Only after the use of IS in a concentration exceeding 0.5 mM in HUVEC, the level of glutathione dropped significantly [20]. These results confirm a significant decrease in the non-enzymatic antioxidant system studied in our work in RBC incubated with IS at a concentration of 1.0 mM. In addition, a negligible decrease in the CAT activity and a statistically significant increase in the SOD activity in the hemolysate were observed.

The obtained results show that IS significantly contributes to the reduction in plasma CAT activity and the increase in the SOD activity in erythrocytes. Moreover, no changes in the CAT activity in RBC incubated with IS were found. These results are in line with the previously published studies on the activity of CAT and SOD in lymphocytes treated with IS [10]. However, a study of the effects of IS on intestinal epithelial cells (IECs) provided information about a reduction in SOD expression in these cells [11]. It is likely that IS may have a different effect on the intestinal epithelial cells than it has on the erythrocytes. This is probably because intestinal epithelial cells are in constant contact with a high concentration of IS in the intestines before IS enters the bloodstream. These results do not have to be mutually exclusive as the functions and metabolism of the tested cells are different. A lower level of CAT activity in plasma was observed in patients with diseases related to oxidative stress, such as diabetes mellitus (type I and II), neurodegenerative diseases, cancer, and anemia [21]. In turn, the increased oxidative stress is compensated by the elevated activity of SOD and CAT during aging in human erythrocytes. Generally, this may be a manifestation of increased ROS/RNS production during human aging [22].

Uremic toxins often exhibit a different mechanism of toxicity toward biomolecules. Our studies analyze the effect of IS in systems devoid of other uremic toxins and investigate the defense mechanisms of RBC and plasma directed toward counteracting the toxicity of this specific uremic toxin. Hence, no significant changes in the level of TBARS or the carbonyls and thiol groups in plasma were observed. Other studies show significant changes in these plasma parameters observed in patients with CKD, where the protein damage was caused by multiple uremic toxins [23,24].

Our results show a decline in the activity of the antioxidant systems and oxidative stress that occurs in RBC. Less significant changes in the examined parameters were observed in the plasma, where a tendency towards a decrease in the thiol groups and a slight rise in the parameters of oxidative stress (the carbonyl compounds and TBARS results) were found. In plasma, the only statistically significant parameter was the decrease in the CAT activity. These results may indicate that plasma, which is directly exposed to the IS effect, is better protected than RBC, despite their high antioxidant potential. However, a significant decrease in the amino groups, both in RBC and plasma, was observed, which could be related to the direct actions of IS. Consequently, this may lead to changes in the properties and functions of many plasma proteins and enzymes, which can trigger the development of other pathologies accompanying CKD. It has also been suggested that IS can be one of the major contributors to the shortening of RBC lifespan in patients with CKD [25,26], and our results support this suggestion.

Chronic renal failure and the accumulation of uremic toxins may accompany other pathologies and is not limited to kidneys only. It was found that, in chronic kidney disease, the intestinal microbial community may be altered. This, in turn, may cause the development of intestinal inflammation; changes in epithelial barrier activity; and a consequent oxidative stress injury to the kidneys, cardiovascular, and endocrine systems [27]. It has been shown that oxidative stress-related redox imbalance caused by the uremic toxins is linked to vascular and rheological complications such as cardiovascular disease, renal function decline, uremic bone disease, muscle wasting, and renal anemia [28]. In addition, IS is believed to be associated with cardiorenal syndrome [29,30].

## 4. Materials and Methods

### 4.1. Chemicals

The following chemicals were purchased from Sigma Chemical Co. (St.Louis, MO, USA): 2,4-dinitrophenylhydrazine (DNPH), 2,4,6-trinitrobenzene sulfonic acid (TNBS), 5,5′-dithiobis(2-nitrobenzoic acid) (DTNB), 4,4′-dithiodipyridine, 2,4,6-tripyridyl-s-triazine (TPTZ), 2,2-diphenyl-1-picrylhydrazyl (DPPH), and adrenalin. All other reagents of analytical purity were obtained from POCH S.A. (Gliwice, Poland).

### 4.2. Hemolysate and Plasma Preparation

Experiments were conducted on RBC hemolysate and plasma isolated from the human blood buffy coat obtained from the blood bank in Lodz, Poland. During centrifugation of blood buffy coat, plasma and other blood morphotic elements were separated. The RBCs were washed three times with PBS (10 mM phosphate-buffered saline, pH 7.4). Erythrocytes were separately incubated in phosphate-buffered saline (pH 7.4) (hematocrit of 50%), and so was plasma for 24 h at 37 °C with IS at a final concentration of either 0.2 mM or 1 mM. After incubation, hemolysate from RBC was prepared by the Drabkin method with the addition of water [31]. Total hemoglobin (Hb) was estimated as cyanmethemoglobin using Drabkin’s reagent and absorbance measurements at 546 nm [31]. The concentration of plasma proteins was determined spectrophotometrically with Folin and Ciocalteu’s phenol reagent, according to the method outlined by Lowry et al. [32]. Hemolysate and plasma were used for future experiments.

In the manuscript, *n*-numbers represent samples from different donors.

### 4.3. Carbonyl Group Content

The protein carbonyl content in plasma and hemolysate was determined with 2,4-dinitrophenylhydrazine (DNPH) [33]. In this method, DNPH reacts with the protein carbonyl groups, leading to the formation of protein-conjugated dinitrophenylhydrazones (DNP), which are optically active at 360 nm. The content of the carbonyl compounds was calculated using the millimolar absorption coefficient (22 mmol^−1^·cm^−1^) and expressed as nmol/mg plasma protein or Hb.

### 4.4. Amino Group Content

The concentration of the free amino group in plasma and hemolysate was estimated using the method described by Crowell et al. [34]. The reaction of 2,4,6-trinitrobenzene sulfonic acid (TNBS) with amines generates a colored product that can be readily measured at 335 nm. The concentration of the amino group was estimated based on the standard curve prepared for different concentrations of homocysteine (in the range of 0–300 µM) and was calculated as nmol/mg plasma protein or Hb.

### 4.5. Thiol Group Content

The concentration of the free thiols in plasma proteins was determined using Ellman’s reagent ((5,5′-dithiobis(2-nitrobenzoic acid); DTNB) [35]. During the reaction with free thiol groups, 2-nitro-5-thiobenzoate (NTB), optically active at 412 nm, is formed.

The concentration of the free thiols in hemolysate was determined using 4,4′-dithiodipyridine [36]. 2-thiopyridone, formed in the reaction of thiols with 4,4′-dithiodipyridine, was measured at 324 nm.

The concentration of the thiol groups, for both methods, was estimated based on the standard curves prepared for different concentrations of reduced glutathione (in the range of 0–0.5 mM) and was calculated as nmol/mg plasma protein or Hb.

### 4.6. Thiobarbituric-Acid-Reactive Substances Content

Plasma and hemolysate lipid peroxidation was assayed by determining the interaction of thiobarbituric acid (TBA) with the breakdown product of lipid peroxidation under acid pH conditions. The pink product of the reaction was determined at 535 nm [37]. The TBARS concentration was calculated using the millimolar absorption coefficient (156 mmol^−1^·cm^−1^) and expressed as nmol/mg plasma protein or Hb.

### 4.7. Total Non-Enzymatic Antioxidant Capacity

The total non-enzymatic antioxidant capacity (NEAC) of plasma and hemolysate was determined using two independent methods. The first one is based on the reduction of ferric-2,4,6-tripyridyl-s-triazine (Fe [III]-TPTZ) complex by cellular antioxidants to an optically active at 593 nm ferrous [Fe-(II)] complex [38]. The second one is based on the reduction of a colored solution of 2,2-diphenyl-1-picrylhydrazyl (DPPH) by cellular antioxidants. The decrease in the color intensity measured at 517 nm is inversely proportional to the level of antioxidants [39].

Calibration curves were prepared for both methods using different concentrations of Trolox (0–1000 µmol/L). The results were expressed as nmol of Trolox equivalents per milligram of plasma protein or Hb.

### 4.8. Catalase Activity

Catalase (CAT) activity in plasma and hemolysate was estimated according to the method described by Aebi [40]. The decrease in absorbance of hydrogen peroxide was measured at 240 nm. The results were expressed in units of CAT activity per milligram of plasma protein or Hb (U/mg protein/min), where 1 U of CAT is defined as the amount of enzyme needed to decompose 1 μmol of hydrogen peroxide per minute.

### 4.9. Superoxide Dismutase Activity

Superoxide dismutase (SOD) activity in hemolysate was estimated according to the method based on the ability of SOD to inhibit adrenalin self-oxidation to adrenochrome in alkaline conditions (pH 10.2) [41]. The increase in absorbance of adrenochrome was measured at 480 nm. SOD activity was calculated as U/mg Hb/min.

### 4.10. Statistical Analysis

All data were expressed as mean ± standard deviation. The obtained results were analyzed as paired data (control, IS—0.2 mM and 1.0 mM from an independent donor). Then, for each parameter, the normality of data was tested using the Shapiro–Wilk test, and homogeneity of variance was verified with Levene’s test. The significance of the differences between the groups was estimated by a one-way ANOVA for repeated measures and by Tukey’s post hoc multiple comparisons test. Statistical significance was accepted at *p* < 0.05 at the least. The statistical analysis was performed using Statistica v. 13.3 (StatSoft Polska, Krakow, Poland).

## 5. Conclusions

The presented study shows that IS leads to a decrease in total non-enzymatic antioxidant capacity and a decrease in thiol and amino groups while also introducing a tendency for oxidative damage (higher carbonyl compounds and TBARS) in RBC and plasma. These changes in the tested parameters were greater in RBC than in plasma, which may indicate better antioxidant protection of plasma. In addition, the observed decrease in IS-induced amino groups in RBC and plasma may cause modifications of proteins and enzyme structure or activity. These results also confirm previous research data, where IS led to a decrease in the lifespan of RBC in the bloodstream of CKD patients.

## Data Availability

Not applicable.

## Abbreviations

CAT	catalase.
CKD	chronic kidney disease.
DPPH	2,2-diphenyl-1-picrylhydrazyl.
Hb	hemoglobin.
HUVEC	human umbilical vein endothelial cells.
IS	indoxyl sulfate.
NEAC	non-enzymatic antioxidant capacity.
RBC	erythrocytes.
ROS	reactive oxygen species.
SOD	superoxide dismutase.
TBARS	thiobarbituric acid reactive substances.
TPTZ	ferric-2,4,6-tripyridyl-s-triazine.
